# Can I Trust This Person? Evaluations of Trustworthiness From Faces and Relevant Individual Variables

**DOI:** 10.3389/fpsyg.2022.857511

**Published:** 2022-05-10

**Authors:** Josefa N. S. Pandeirada, Natália Lisandra Fernandes, Mariana Madeira, Patrícia I. Marinho, Marco Vasconcelos

**Affiliations:** ^1^William James Center for Research, University of Aveiro, Aveiro, Portugal; ^2^Department of Education and Psychology, University of Aveiro, Aveiro, Portugal; ^3^SASUA, University of Aveiro, Aveiro, Portugal

**Keywords:** trustworthiness, human faces, Portuguese, evaluations, individual variables

## Introduction

Facial appearance plays a decisive role during impression formation (e.g., Hassin and Trope, 2000). Drawing trait inferences from faces occurs extremely fast (i.e., within 100 milliseconds; e.g., Willis and Todorov, 2006) and appears early in childhood. This seems to be particularly true for trustworthiness judgments which develop earlier than other judgments and with high levels of consistency with judgments made by adults (Cogsdill et al., 2014). High agreement is commonly found in facial trustworthiness ratings (Todorov, 2008). Because this dimension is highly correlated with other judgments, Todorov (2008) proposed that facial trustworthiness “may reflect the general evaluation of the face” (p. 210). Furthermore, trustworthiness judgments tend to guide peoples' decisions, even when other more diagnostic cues are available, suggesting that these judgments might be somewhat imprecise (e.g., Jaeger et al., 2020; however, see Chang et al., 2010). To underscore the relevance of these judgments *per se*, as well as their involvement in more ecologically relevant tasks, we briefly mention studies based on targeted judgments of facial trustworthiness and studies relying on actual behaviors.

In the criminal context, for example, Flowe (2012) reported that faces perceived as untrustworthy were rated as appearing more criminal, as compared to faces considered trustworthy. In a related work, Porter et al. (2010) revealed that, when dealing with weak or ambiguous evidence, untrustworthy-looking defendants were more likely to receive a guilty verdict. Additionally, the jurors' confidence levels in such decisions were higher for individuals rated as less trustworthy. Accordingly, untrustworthy-looking defendants received harsher criminal sentences (Wilson and Rule, 2015), and experienced less leniency (Jaeger et al., 2020).

Financial behavior is also impacted by trustworthiness inferences. Individuals invest more money in trust games when their opponents look trustworthy (vs. untrustworthy) (Chang et al., 2010; Kroneisen et al., 2021) and cooperate less with untrustworthy-looking opponents (Kroneisen et al., 2021). Duarte et al. (2012) also showed that more trustworthy-looking individuals had higher loan approval rates and better credit scores.

Social interactions are also informed by trustworthiness perceptions. For example, individuals with trustworthy faces are more likely to be accepted in groups than those with untrustworthy faces (Tracy et al., 2020). In some cultures, inferences related to trustworthiness are also valued in future leaders and predictive of electoral success (Rule et al., 2010).

Several individual characteristics seem to influence one's behaviors and evaluations of trustworthiness. For example, an individual's trust attitudes relate with his/her evaluations of others' trustworthiness, as well as with his/her trust behaviors toward them. Whitener et al. (1998) reported that individuals with a high propensity to trust in others expect more trustworthy (or cooperative) behaviors from them in real interactions (e.g., Gill et al., 2005). Given that participants' trust attitudes have been measured mostly through self-report questions (e.g., Glaeser et al., 2000; Ben-Ner and Halldorsson, 2010), it would be interesting to explore if higher trust attitudes relate with higher facial evaluations of trustworthiness.

Even though the evidence is scarce, some studies suggest that the sex of the rater may play a role in trustworthy judgments. For instance, some studies have reported that women rate trustworthy-looking faces as significantly more trustworthy than men; furthermore, women assigned higher trustworthiness to female than to male faces (Mattarozzi et al., 2015). Still, behavioral studies have returned mixed evidence. Whereas some found no sex differences regarding trust in others (see Croson and Buchan, 1999; Dreber and Johannesson, 2008), others have reported that females are more reluctant to trust others (e.g., Glaeser et al., 2000; Ben-Ner and Halldorsson, 2010). Nevertheless, in both types of studies, females tended to be evaluated as more trustworthy than males (e.g., Croson and Buchan, 1999; Mattarozzi et al., 2015). Therefore, one would expect female (vs. male) faces to be perceived as more trustworthy by both sex raters, but predictions regarding the influence of the rater's sex on the evaluations are not clear-cut; the data reported here might contribute to this ongoing debate.

Trust behaviors toward others seem to increase with age (e.g., Alesina and La Ferrara, 2002). Older adults tend to trust more frequently in people with a reputation of being untrustworthy than young adults (e.g., Bailey et al., 2015; although see Sutter and Kocher, 2007), which makes them more vulnerable to subsequent exploitation and fraud (e.g., Castle et al., 2012). Accordingly, studies based on facial trustworthiness evaluations report that even though there is a high agreement on the ratings provided by older and young adults, the former tend to perceive faces as more trustworthy (Castle et al., 2012; Cassidy et al., 2019). These studies used a relatively small number of faces and collected a relatively small number of ratings per age group. We provide ratings for a much larger set of stimuli and from a larger and more heterogeneous sample of participants; our data should contribute to the investigation of this topic.

Several studies have also reported a positive relation between trusting behaviors and level of education: the higher the level, the higher the trust in others (Holmberg and Rothstein, 2017). Relatedly, Knack and Zak (2003) noted that a society that raises the level of education concomitantly promotes trust in others. Whether the same relation would be obtained in facial trustworthiness evaluations is an open question and our data should speak to that.

Regarding the influence of marital status on the perception of facial trustworthiness, data are scarce. At the behavioral level, Alesina and La Ferrara (2002) reported that although marital status *per se* had no effect on trust, when the divorce or separation processes were somehow traumatic, trust in others declined substantively. Glaeser et al. (2000), in contrast, reported that married individuals (vs. other types of relationship status) were more trusting. We present individual information on marital status in order to broaden this still limited literature.

As briefly reviewed, facial trustworthiness impacts various domains and much remains to be known about the role of several individual variables. Here, we provide facial trustworthiness evaluations from the Portuguese population for a large set of faces (*N* = 231) gathered from different international databases. Data were collected online and in the laboratory, thus contributing to the ongoing discussion regarding the reliability of online collected data (e.g., Walter et al., 2019). Finally, we present data on individual variables that might influence the perception of facial trustworthiness and/or trust behaviors: sex, age, years of education, marital status, self- and others-perceived trustworthiness, and the participants' trust attitudes. To our best knowledge, only one study conducted with the Portuguese population reports trustworthiness evaluations for faces; yet, it was limited to a specific database (not included in our work) and it did not cover the variables explored here (cf. Ramos et al., 2016).

## Method

### Participants

#### Online Sample

A questionnaire was completed online by 822 participants. The following elimination criteria were implemented: non-Portuguese participants (as we sought to collect data from the Portuguese population; *n* = 68); randomization errors in the questionnaire (*n* = 16); and, underaged participants (*n* = 1). The final sample consisted of 737 participants, aged between 18 and 78 years (*M* = 35.0, *SD* = 13.0). The questionnaire was not fully completed by 314 other participants.

#### Laboratory Sample

The same online questionnaire was completed in a laboratory setting by 119 valid participants with a mean age of 21 years (*SD* = 4.1; range: 18–53). Eleven other participants were excluded for being non-Portuguese (*n* = 8) or due to questionnaire randomization errors (*n* = 3).

Table 1 reports a complete characterization of the two samples regarding sex, age group, marital status, and years of education.

**Table 1:** Characterization of the sample regarding sex, age group, marital status, and years of education.

	**Online sample**		**Laboratory sample**	
	***N***	**Percentage**	***N***	**Percentage**
**Sex**				
Female	483	65.5	82	68.9
Male	254	34.5	37	31.1
**Age group**				
Young adults (18–29 years)	322	43.7	114	95.8
Middle-aged adults (30–49 years)	293	39.8	4	3.4
Older adults (≥50 years)	122	16.6	1	0.8
**Marital Status**				
Single	393	53.3	116	97.5
Married	282	38.3	2	1.7
Divorced	55	7.5	1	0.8
Widowed	7	0.9	0	
**Education in years**				
≤ 12 years of education	106	14.4	23	19.3
>12 years of education	629	85.3	96	80.7

### Material

We used 231 frontal-view, colored young adult facial photographs (126 males and 105 females), displaying a neutral facial expression and direct eye gaze. Two of the authors selected existing face databases that complied with the following: (a) they contained faces similar to those of the Portuguese population; (b) photographs were taken under controlled and similar conditions (e.g., illumination setting and uniform background); (c) individuals used a standard t-shirt and removed jewelry, glasses, and makeup; and, (d) faces were of young adults. Faces from five databases were selected based on these criteria: (1) Karolinska Directed Emotional Faces (KDEF; Goeleven et al., 2008); (2) Warsaw Set of Emotional Facial Expression Pictures (WSEFEP; Olszanowski et al., 2014); (3) Radboud Facial Database (RaFD; Langner et al., 2010); (4) FACES Database (Ebner et al., 2010); and, (5) Amsterdam Dynamic Facial Expression Set (ADFES; van der Schalk et al., 2011). Proper permission was secured to use these databases and, when necessary, to edit the pictures. Edition of the pictures was as described in Pandeirada et al. (2020) and aimed to obtain a more homogeneous set of stimuli^1^. The final distribution of stimuli by database and the specific faces used in the study are presented in the “Read me first” worksheets of the files available on OSF (https://osf.io/ckmd4/?view_only=83c5fe29850847868cad3a09de8ace2d). Each participant evaluated only 50 faces that were pseudo-randomly selected from the total set of stimuli with the following constraints: (1) the same number of female (*n* = 25) and male faces (*n* = 25) was presented in each questionnaire; and, (2) the number of faces selected from a given database was proportionally similar for all databases. We decided to ask each participant to rate only a subset of the total set of faces to shorten the task and thus increase the likelihood of task completion.

### Procedure

A questionnaire was administered using the software LimeSurvey. The questionnaire, as well as the data, were housed in a local server at the University of Aveiro. For the online data collection, a brief description of the questionnaire and its electronic link was disseminated via electronic mail. As we were aiming to collect a large number of ratings per face, the link was sent to several organizations of various types across the country (e.g., universities, professional schools, industrial organizations, technological organizations) with a request to share the link with their collaborators. For the laboratory data collection, participants were recruited from three Portuguese public Universities. All participants were required to be at least 18 years old and of Portuguese nationality; no other exclusion criteria were presented. Data collection occurred between June of 2014 and May of 2015.

The questionnaire started with a brief description of the study and an informed consent request. If no consent was given, participants were thanked and the program ended; otherwise, the program moved on to collect information on sex, age, nationality, marital status, and years of education.

The trustworthiness-rating instructions then informed participants they would be shown faces sequentially and should: “observe each face and indicate how trustworthy each face is to you, that is, to what extent you could trust this person”. The 7-point rating scale was also described, indicating that 1 corresponded to “not trustworthy at all”, and 7 to “very trustworthy”. The task was self-paced but participants were instructed to respond quickly and to rely on their “gut feeling”. They were also told that their evaluations represented their personal view and that there were no correct or incorrect responses. Faces were then displayed, one at a time, at the center of a white background screen with the response scale bellow; this consisted of a series of radio buttons along with the labels for the values 1 and 7. Responses were mandatory and given through the selection of their chosen number. Each picture was preceded by a 1,000-ms fixation cross and followed by a 500-ms blank screen. A different presentation order was used for each participant.

After rating all the faces, participants were asked to rate their own trustworthiness (i.e., how trustworthy they considered themselves to be) and how trustworthy they thought other people would rate them. The rating procedure described above was followed here except that a shadow face represented the participant's face. Then, to measure trust attitudes as in previous studies (e.g., Ben-Ner and Halldorsson, 2010), participants answered the following questions: (1) “Generally speaking, would you say that most people can be trusted, or that you cannot be too careful when dealing with people?”; (2) “Which of the following statements best reflects your view? I will not trust a person until there is clear evidence that he or she can be trusted, or I will trust a person until I have clear evidence that he or she can't be trusted”; (3) “On a scale from 1 to 6 where 1 is “Relatively cautious” and 6 is “Relatively trusting”, how would you describe your interactions with other people?”. The questionnaire ended here for male participants. Additional information, of no relevance for the current study, was collected from female participants^2^. A final appreciation message was presented at the end. All procedures complied with the applicable aspects of the 1964 Helsinki declaration and its later amendments.

## Data Set Characterization

Due to the pseudo-random selection of the 50 faces presented in each questionnaire, a variable number of participants contributed to the rating of each face. On average, each face was rated by 159.52 participants from the online sample (*SD* = 20.55; range 117–215), and by 25.76 participants from the laboratory sample (*SD* = 5.35; range 13–38).

The data, collected both online and in the laboratory, are made available through OSF (https://osf.io/ckmd4/?view_only=83c5fe29850847868cad3a09de8ace2d). The responses given by each participant and the data organized by item are in files “Trustworthiness_Subject Data.xlsx” and “Trustworthiness_Item Data.xlsx”, respectively. In the latter, we present the number of ratings that contributed to the mean rating (and corresponding SD) of each face. Item-based data are also presented separately for the online and laboratory samples, and according to different variable conditions as described below. We do not present data when the mean number of observations for the face was below five as these would be rather unrepresentative; these exceptions are noted in the corresponding spreadsheets. The item data file includes the following tabs:

(1) Read me first: Describes what can be found in each of the remaining tabs. The number of faces from each database that was presented in each questionnaire is shown at the bottom of this tab;
(2) Overall Data: Presents the overall trustworthiness ratings (means and SD) for each face;
(3) Sex: Presents information for each face according to the sex of the participant;
(4) Age Group: Data are provided separately for three age groups according to the age of the rater (as in McLellan and McKelvie, 1993): young-adult raters, middle-aged raters and old raters;
(5) Marital Status: Ratings are provided according to the participants' relationship situation; data were broken down depending on whether the participant was single, married, or divorced;
(6) Years of Education: Ratings are presented broken down by the number of years of education participants have (≤12 or >12);
(7) Self and Others Evaluation: The participants' responses on self and other evaluations were recoded to create three groups: low (ratings of 1–2), average (ratings of 3–5), and high trustworthiness (ratings of 6–7).

To give the reader a better sense of the obtained data and their potential use, we report in Table 2 the mean number of ratings and mean trustworthiness values per face and broken down by different variables.

**Table 2:** Mean number of responses and mean trustworthiness ratings, and corresponding standard deviations (SD), for all faces and separately for the female and male faces.

	**All faces**				**Female faces**				**Male faces**			
	**Mean number of responses** **(SD)**		**Mean trustworthiness** **(SD)**		**Mean number of responses** **(SD)**		**Mean trustworthiness** **(SD)**		**Mean number of responses** **(SD)**		**Mean trustworthiness** **(SD)**	
**Raters**	**Online**	**Lab**	**Online**	**Lab**	**Online**	**Lab**	**Online**	**Lab**	**Online**	**Lab**	**Online**	**Lab**
Full sample	159.52 (20.55)	25.76 (5.35)	3.87 (0.53)	3.68 (0.66)	175.48 (15.90)	28.33 (4.93)	4.07 (0.52)	3.92 (0.66)	146.23 (13.21)	23.61 (4.71)	3.71 (0.47)	3.48 (0.60)
**Sex**												
Female	104.55 (14.36)	17.75 (4.05)	3.84 (0.56)	3.69 (0.72)	115.00 (11.23)	19.52 (3.83)	4.05 (0.56)	3.97 (0.73)	95.83 (10.31)	16.27 (3.63)	3.66 (0.51)	3.45 (0.63)
Male	54.98 (9.18)	8.32 (2.23)	3.94 (0.49)	3.67 (0.71)	60.48 (8.80)	8.86 (2.24)	4.10 (0.49)	3.79 (0.68)	50.40 (6.63)	7.82 (2.10)	3.81 (0.45)	3.57 (0.72)
**Age group**												
Young adults (18-29 years)	69.70 (10.56)	24.68 (5.16)	3.81 (0.60)	3.68 (0.67)	76.67 (8.59)	27.14 (4.66)	4.04 (0.58)	3.92 (0.67)	63.89 (8.31)	22.62 (4.63)	3.62 (0.54)	3.47 (0.60)
Middle-aged adults (30-49 years)	63.42 (9.70)	-	3.92 (0.50)	-	69.76 (8.72)	-	4.09 (0.51)	-	58.13 (6.93)	-	3.78 (0.45)	-
Older adults (≥50 years)	26.41 (5.54)	-	3.92 (0.52)	-	29.05 (5.35)	-	4.07 (0.51)	-	24.21 (4.68)	-	3.80 (0.50)	-
**Marital status**												
Single	85.06 (12.15)	25.11 (5.23)	3.84 (0.57)	3.68 (0.67)	93.57 (9.49)	27.62 (4.73)	4.07 (0.56)	3.92 (0.66)	77.98 (9.23)	23.02 (4.68)	3.66 (0.52)	3.47 (0.61)
Married	61.04 (9.67)	-	3.95 (0.50)	-	67.14 (8.25)	-	4.11 (0.51)	-	55.95 (7.62)	-	3.81 (0.44)	-
Divorced	12.02 (3.15)	-	3.75 (0.64)	-	13.18 (2.99)	-	3.91 (0.56)	-	11.04 (2.95)	-	3.62 (0.67)	-
**Years of education**												
≤12 years of education	22.94 (5.02)	6.43 (1.53)	3.65 (0.56)	3.63 (0.83)	25.24 (4.64)	6.43 (1.39)	3.82 (0.53)	3.87 (0.86)	21.03 (4.50)	6.44 (1.70)	3.52 (0.54)	3.34 (0.70)
>12 years of education	136.15 (17.86)	20.78 (4.76)	3.91 (0.53)	3.72 (0.67)	149.76 (13.86)	22.86 (4.41)	4.11 (0.53)	3.94 (0.67)	124.80 (11.89)	19.05 (4.35)	3.75 (0.48)	3.53 (0.62)

*Data are also presented broken down by sex, age group, marital status, and number of years of education. These data are also broken down by sample (online and laboratory samples)*.

*Data on some variables are not presented as the overall mean number of ratings was lower than five (see the Datafiles for more details)*.

## Suggestions for Future Use of This Data Set

Trustworthiness judgments are one of the cornerstones of trait inferences and play an important adaptive function (Todorov et al., 2008). It is one of the most important aspects people consider when evaluating faces, although some variation seems to exist across different countries (Jones et al., 2021). We report trustworthiness evaluations for a large set of face images. We also provide information on several variables of potential interest to trustworthiness inferences hoping to inspire readers to explore the potential of this dataset, and thus help complement past research and shed light into future research avenues.

Regarding data collection, we sought to contribute to the debate concerning the reliability of data collected online and in the laboratory. To preview, a brief inspection of our data revealed an excellent agreement between these two datasets from young adults [Intra Class Correlation (ICC) = .92], similarly to what was reported in other studies (e.g., Maeder et al., 2018). We should note that we only used young-adult faces and our sample procedures did not guarantee Portuguese representative samples; these are limitations of our study. For the online data collection, the questionnaire was disseminated through organizations from across the country, whereas the laboratory data were gathered from three restricted Universities. Still, the data from these two samples are in high agreement and we provide a quite large number of data points per face from the online sample.

Some literature reports agreement on trustworthiness evaluations across observers and cultures (e.g., Sutherland et al., 2018, 2020). An exploratory analysis revealed that our ratings agree strongly with those obtained in the Netherlands by Jaeger (2020) for the RaFD faces (ICC = .95 and .92, with our young-adults online and laboratory data, respectively). Good agreement was also achieved between our young-adult laboratory ratings and those collected in Spain by Aguado et al. (2011) for the KDEF faces (ICC = .78), in spite of the fact that faces were presented in grayscale in that study. Considering these exploratory results, we are fairly confident that our data could reliably be used by researchers from other countries. Nonetheless, more comparisons could be made with other studies that provide data for some of the stimuli here used, while considering methodological differences that likely affect the results. For example, trustworthiness ratings of the KDEF faces were provided by Gutiérrez-García et al. (2019), Sutton et al. (2019), and Sutherland et al. (2017); however, they all used faces displaying different emotions and, in the latter, also in different viewpoints.

Inferences from faces rarely occur for a single dimension and tend to influence our behavior in a concerted way purportedly to activate the most adaptive responses (Todorov et al., 2008). In a previous study, we have made available ratings of attractiveness for 96.5% of the faces included in the present report. These combined sets of data will allow researchers to select stimuli and/or analyze the data while considering these two important dimensions. In all, the current data add to the build-up of an integrated dataset that should be of great use for researchers from various areas.

## Data Availability Statement

The datasets presented in this study can be found online in the following repository: https://osf.io/ckmd4/?view_only=83c5fe29850847868cad3a09de8ace2d.

## Ethics Statement

Ethical review and approval was not required for the study on human participants in accordance with the local legislation and institutional requirements in place at the time the study was conducted. The participants provided their written informed consent to participate in this study. All procedures were in accordance with the 1964 Helsinki declaration and its later amendments, in particular with the following aspects: participation in the project added no risk or burden to participants, the benefits of the project clearly outlined its potential risks, written informed consent was obtained from all participants, participation was voluntary, and data were managed and are presented in a manner that ensures the participants' anonymity.

## Author Contributions

JP and NF developed the idea and procedures to collect the data. JP coordinated the data acquisition. NF managed the data collection process and provided preliminary analysis. MM and PM contributed to the writing of the Introduction and Discussion. All contributed to the writing of the manuscript which was then discussed, reviewed, and commented along with MV. All authors approved the final version of this manuscript.

## Funding

The Portuguese Foundation for Science and Technology provided support to JP (Research and Investigator Grant, ref. IF/00058/2012/CP0172/CT0002, PTDC/MHC-PCN/5274/2012, and CEECIND/01914/2017), to NF and PM (Research Grant PTDC/MHC-PCN/5274/2012). MM was supported by a research grant awarded by the University of Aveiro. Support was also attained through the WJCR (UID/04810/2020).

## Conflict of Interest

The authors declare that the research was conducted in the absence of any commercial or financial relationships that could be construed as a potential conflict of interest.

## Publisher's Note

All claims expressed in this article are solely those of the authors and do not necessarily represent those of their affiliated organizations, or those of the publisher, the editors and the reviewers. Any product that may be evaluated in this article, or claim that may be made by its manufacturer, is not guaranteed or endorsed by the publisher.

## Footnotes

^1^Six of the photographs from the original datasets were excluded for one of the following: (a) the top of the head was not visible; (b) facial characteristics were too different from the Portuguese population; or, (c) we were unable to edit the photograph to make it homogenous with the remaining stimulus set.

^2^Female participants provided information about their menstrual cycle (average duration, date of last menstrual cycle, and use of oral contraceptives).

## References

[B1] Aguado L. Román F. J. Fernández-Cahill M. Diéguez-Risco T. Romero-Ferreiro V. (2011). Learning about faces: effects of trustworthiness on affective evaluation. Span. J. Psychol. 14, 523–534. 10.5209/rev_SJOP.2011.v14.n2.1 22059299

[B2] Alesina A. La Ferrara E. (2002). Who trusts others? J. Public Econ. 85, 207–234. 10.1016/S0047-2727(01)00084-6

[B3] Bailey P. E. Szczap P. McLennan S. N. Slessor G. Ruffman T. Rendell P. G. (2015). Age-related similarities and differences in first impressions of trustworthiness. Cogn. Emot. 30, 1–10. 10.1080/02699931.2015.1039493 26016678

[B4] Ben-Ner A. Halldorsson F. (2010). Trusting and trustworthiness: what are they, how to measure them, and what affects them. J. Econ. Psychol. 31, 64–79. 10.1016/j.joep.2009.10.001

[B5] Cassidy B. S. Boucher K. L. Lanie S. T. Krendl A. C. (2019). Age effects on trustworthiness activation and trust biases in face perception. J Gerontol B Psychol Sci Soc Sci. 74, 87–92. 10.1093/geronb/gby062 29846711 PMC6294231

[B6] Castle E. Eisenberger N. I. Seeman T. E. Moons W. G. Boggero I. A. Grinblatt M. S. . (2012). Neural and behavioral bases of age differences in perceptions of trust. Proc. Natl. Acad. Sci. U. S. A. 109, 20848–20852. 10.1073/pnas.1218518109 23213232 PMC3529090

[B7] Chang L. J. Doll B. B. van't Wout M. Frank M. J. Sanfey A. G. (2010). Seeing is believing: trustworthiness as a dynamic belief. Cogn. Psychol. 61, 87–105. 10.1016/j.cogpsych.2010.03.001 20553763

[B8] Cogsdill E. J. Todorov A. T. Spelke E. S. Banaji M. R. (2014). Inferring character from faces: A developmental study. Psychol. Sci. 25, 1132–1139. 10.1177/0956797614523297 24570261 PMC5580985

[B9] Croson R. Buchan N. (1999). Gender and culture: International experimental evidence from trust games. Am. Econ. Rev. 89, 386–391. 10.1257/aer.89.2.386

[B10] Dreber A. Johannesson M. (2008). Gender differences in deception. Econ. Lett. 99, 197–199. 10.1016/j.econlet.2007.06.027

[B11] Duarte J. Siegel S. Young L. (2012). Trust and credit: The role of appearance in peer-to-peer lending. Review of Financial Studies, 25(8), 2455–2483. 10.1093/rfs/hhs071

[B12] Ebner N. C. Riediger M. Lindenberger U. (2010). FACES — A database of facial expressions in young, middle-aged, and older women and men: development and validation. Behav. Res. Methods. 42, 351–362. 10.3758/BRM.42.1.351 20160315

[B13] Flowe H. D.. (2012). Do characteristics of faces that convey trustworthiness and dominance underlie perceptions of criminality? PloS ONE. 7:e37253. 10.1371/journal.pone.0037253 22675479 PMC3366989

[B14] Gill H. Boies K. Finegan J. E. McNally J. (2005). Antecedents of trust: establishing a boundary condition for the relation between propensity to trust and intention to trust. J Bus Psychol. 19, 287–302. 10.1007/s10869-004-2229-8

[B15] Glaeser E. L. Laibson D. I. Scheinkman J. A. Soutter C. L. (2000). Measuring trust. Q. J. Econ. 115, 811–846. 10.1162/003355300554926

[B16] Goeleven E. De Raedt R. Leyman L. Verschuere B. (2008). The Karolinska directed emotional faces: a validation study. Cogn. Emot. 22, 1094–1118. 10.1080/0269993070162658229312053

[B17] Gutiérrez-García A. Beltrán D. Calvo M. G. (2019). Facial attractiveness impressions precede trustworthiness inferences: lower detection thresholds and faster decision latencies. Cogn. Emot. 33, 378–385. 10.1080/02699931.2018.1444583 29482469

[B18] Hassin R. Trope Y. (2000). Facing faces: Studies on the cognitive aspects of physiognomy. J. Pers. Soc. Psychol. 78, 837–852. 10.1037/0022-3514.78.5.837 10821193

[B19] Holmberg S. Rothstein B. (2017). Trusting other people. J. Public Affairs. 17, 1–8. 10.1002/pa.1645

[B20] Jaeger B. Todorov A. T. Evans A. M. van Beest I. (2020). Can we reduce facial biases? Persistent effects of facial trustworthiness on sentencing decisions. J. Exp. Soc. Psychol. 90, 1–12. 10.1016/j.jesp.2020.104004

[B21] Jaeger B.. (2020). Trait ratings for the Radboud Faces Database. Available online at: https://psyarxiv.com/cf5ad/ (accessed January, 2021).

[B22] Jones B. C. DeBruine L. M. Flake J. K. Liuzza M. T. Antfolk J. Arinze N. C. . (2021). To which world regions does the valence–dominance model of social perception apply? Nat. Hum. Behav. 5, 159–69. 10.1038/s41562-020-01007-2 33398150

[B23] Knack S. Zak P. J. (2003). Building trust: Public policy, interpersonal trust, and economic development. Supreme Court Econ. Rev. 10, 91–107. 10.1086/scer.10.114713929421463

[B24] Kroneisen M. Bott F. M. Mayer M. (2021). Remembering the bad ones: Does the source memory advantage for cheaters influence our later actions positively? Q. J. Exp. Psychol. 1–17. 10.1177/17470218211007822 33765882

[B25] Langner O. Dotsch R. Bijlstra G. Wigboldus D. H. J. Hawk S. T. van Knippenberg A. (2010). Presentation and validation of the radboud faces database. Cogn. Emot. 24, 1377–1388. 10.1080/02699930903485076

[B26] Maeder E. M. Yamamoto S. McManus L. A. (2018). Methodology matters: Comparing sample types and data collection methods in a juror decision-making study on the influence of defendant race. Psychology, Crime and Law. 24, 687–702. 10.1080/1068316X.2017.1409895

[B27] Mattarozzi K. Todorov A. Marzocchi M. Vicari A. Russo P. M. (2015). Effects of gender and personality on first impression. PLoS ONE. 10, 1–13. 10.1371/journal.pone.0135529 26331610 PMC4557906

[B28] McLellan B. McKelvie S. J. (1993). Effects of age and gender on perceived facial attractiveness. Can. J. Behav. Sci. 25, 135–142. 10.1037/h007879029867620

[B29] Olszanowski M. Pochwatko G. Kuklinski K. Scibor-Rylski M. Lewinski P. Ohme R. K. (2014). Warsaw set of emotional facial expression pictures: a validation study of facial display photographs. Front. Psychol. 5, 1516. 10.3389/fpsyg.2014.01516 25601846 PMC4283518

[B30] Pandeirada J. N. S. Fernandes N. L. Vasconcelos M. (2020). Attractiveness of human faces: Norms by sex, sexual orientation, age, relationship stability, and own attractiveness judgements. Front. Psychol. 11, 419. 10.3389/fpsyg.2020.00419 32231625 PMC7083125

[B31] Porter S. ten Brinke L. Gustaw C. (2010). Dangerous decisions: the impact of first impressions of trustworthiness on the evaluation of legal evidence and defendant culpability. Psychology, Crime and Law. 16, 477–491. 10.1080/10683160902926141

[B32] Ramos T. Oliveira M. Santos A. S. Garcia-Marques L. Carneiro P. (2016). Evaluating young and old faces on social dimensions: Trustworthiness and dominance. Psicologica. 37, 169–185.

[B33] Rule N. O. Ambady N. Adams R. B. Ozono H. Nakashima S. Yoshikawa S. . (2010). Polling the face: Prediction and consensus across cultures. J. Pers. Soc. Psychol. 98, 1–15. 10.1037/a0017673 20053027

[B34] Sutherland C. A. M. Liu X. Zhang L. Chu Y. Oldmeadow J. A. Young A. W. (2018). Facial first impressions across culture: Data-driven modeling of Chinese and British perceivers' unconstrained facial impressions. Pers Soc Psychol Bull. 44, 521–537. 10.1177/0146167217744194 29226785

[B35] Sutherland C. A. M. Rhodes G. Burton N. S. Young A. W. (2020). Do facial first impressions reflect a shared social reality? Br. J. Psychol. 111, 215–232. 10.1111/bjop.12390 30924928

[B36] Sutherland C. A. M. Young A. W. Rhodes G. (2017). Facial first impressions from another angle: How social judgements are influenced by changeable and invariant facial properties. Br. J. Psychol. 108, 397–415. 10.1111/bjop.12206 27443971

[B37] Sutter M. Kocher M. G. (2007). Trust and trustworthiness across different age groups. Games Econ. Behav. 59, 364–382. 10.1016/j.geb.2006.07.006

[B38] Sutton T. M. Herbert A. M. Clark D. Q. (2019). Valence, arousal, and dominance ratings for facial stimuli. Q. J. Exp. Psychol. 72, 2046–2055. 10.1177/1747021819829012 30760113

[B39] Todorov A.. (2008). Evaluating faces on trustworthiness: an extension of systems for recognition of emotions signaling approach/avoidance behaviors. Ann. N. Y. Acad. Sci. 1124, 208–224. 10.1196/annals.1440.012 18400932

[B40] Todorov A. Said C. P. Engell A. D. Oosterhof N. N. (2008). Understanding evaluation of faces on social dimensions. Trends Cogn. Sci. 12, 455–460. 10.1016/j.tics.2008.10.001 18951830

[B41] Tracy R. E. Wilson J. P. Slepian M. L. Young S. G. (2020). Facial trustworthiness predicts ingroup inclusion decisions. J. Exp. Soc. Psychol., 91, 104047. 10.1016/j.jesp.2020.104047

[B42] van der Schalk J. Hawk S. T. Fischer A. H. Doosje B. (2011). Moving faces, looking places: validation of the Amsterdam dynamic facial expression set (ADFES). Emotion. 11, 907–920. 10.1037/a0023853 21859206

[B43] Walter S. L. Seibert S. E. Goering D. O'Boyle E. H. (2019). A tale of two sample sources: Do results from online panel data and conventional data converge? J Bus Psychol. 34, 425–452. 10.1007/s10869-018-9552-y

[B44] Whitener E. M. Brodt S. E. Korsgaard M. A. Werner J. M. (1998). Managers as initiators of trust: An exchange relationship framework for understanding managerial trustworthy behavior. Acad Manage Rev. 23, 513–530. 10.5465/amr.1998.926624

[B45] Willis J. Todorov A. (2006). First impressions. Psychol. Sci. 17, 592–598. 10.1111/j.1467-9280.2006.01750.x 16866745

[B46] Wilson J. P. Rule N. O. (2015). Facial trustworthiness predicts extreme criminal-sentencing outcomes. Psychol. Sci. 26, 1325–1331. 10.1177/0956797615590992 26162847

